# Water Electrolysis Using a Porous IrO_2_/Ti/IrO_2_ Catalyst Electrode and Nafion Membranes at Elevated Temperatures

**DOI:** 10.3390/membranes11050330

**Published:** 2021-04-30

**Authors:** Je-Deok Kim, Akihiro Ohira

**Affiliations:** 1 Research Center for Functional Materials, Functional Clay Materials Group, National Institute for Materials Science (NIMS), 1-1 Namiki, Tsukuba, Ibaraki 305-0044, Japan; 2 Energy Storage Technology Group, Research Institute for Energy Conservation, National Institute of Advanced Industrial Science and Technology (AIST), 1-1-1 Higashi, Tsukuba, Ibaraki 305-8565, Japan; a-oohira@aist.go.jp

**Keywords:** polymer electrolyte, nafion membrane, porous IrO_2_/Ti/IrO_2_ catalyst electrode, water electrolysis, elevated temperature

## Abstract

Porous IrO_2_/Ti/IrO_2_ catalyst electrodes were obtained by coating IrO_2_ on both sides of three types of porous Ti powder sheets (sample 1, sample 2, and sample 3) using different surface treatment methods, and a hydrogen evolution catalyst electrode was obtained by coating Pt/C on carbon gas diffusion layers. A Nafion115 membrane was used as an electrolyte for the membrane electrode assemblies (MEA). Water electrolysis was investigated at cell temperatures up to 150 °C, and the electrical characteristics of the three types of porous IrO_2_/Ti/IrO_2_ catalyst electrodes were investigated. The sheet resistance of sample 1 was higher than those of samples 2 and 3, although during water electrolysis, a high current density was observed due to the nanostructure of the IrO_2_ catalyst. In addition, the structural stabilities of Nafion and Aquivion membranes up to 150 °C were investigated by using small angle X-ray scattering (SAXS). The polymer structures of Nafion and Aquivion membranes were stable up to 80 °C, whereas the crystalline domains grew significantly above 120 °C. In other words, the initial polymer structure did not recover after the sample was heated above the glass transition temperature.

## 1. Introduction

Global warming is driving societies that burn fossil fuels for energy, which produces huge amounts of CO_2_, towards renewable and sustainable energies. In particular, the use of renewable energy, such as solar and wind power, is drawing attention because it does not emit CO_2_. However, the generation of electricity from photovoltaic cells and wind power generators is variable, and large-capacity energy storage systems are required for efficient use. Energy storage methods include physical energy storage (flywheel, compressed-air energy storage (CAES), pumping) and chemical energy storage (redox flow battery (RFB), sodium-sulfur battery (NAS), Li-ion battery (LIB), methane, hydrogen (H_2_)). In order to supply stable renewable energy, systems with high energy densities and conversion efficiencies are required. Hydrogen has a high energy density, is a CO_2_-free energy source, and is suitable for energy storage systems (gas, metal hydride, liquid) [1,2].

Hydrogen can be produced by using fossil fuel reforming, processing industrial byproducts and biomass, using water electrolysis methods, etc. Among these methods, hydrogen production by water electrolysis has been attracting attention in recent years for use in renewable energy and mobile systems [1,2,3]. Polymer electrolyte membrane water electrolysis (PEMWE, cell temperature: 50–80 °C), alkaline water electrolysis (AWE, cell temperature: 60–80 °C), and solid oxide water electrolysis (SOEC, cell temperature: >600 °C) have been reported. PEMWE, in which a proton exchange membrane and a noble metal catalyst electrode are used, has a higher hydrogen production cost (4.1–8.6 €/kg H_2_) than AWE (3.2–5.0 €/kg H_2_) does. However, the low power and high purity characteristics of PEMWE should improve its efficiency [1,2,3,4,5,6]. More than half of the cost of PEMWE is the stack cost [3,4]. However, the catalyst electrode and electrolyte membrane must have lower costs and higher performances. Iridium oxide and ruthenium oxide, which have high corrosion resistances and excellent catalytic activities, are mainly used as catalysts for oxygen evolution reactions, and platinum is mainly used as a catalyst for hydrogen evolution reactions [6,7,8,9,10,11,12,13,14,15,16]. Nafion and Aquivion membranes, which are perfluorosulfonic acid (PFSA) membranes with high proton conductivities, high chemical stabilities, and high mechanical strengths, are often used as electrolyte membranes [16,17,18,19,20,21].

At the same time, to increase the efficiency of PEMWE, the operating temperature (100–200 °C) must be increased. Electrolysis at elevated temperatures has kinetic and thermodynamic advantages for catalytic electrodes in water splitting [3,19]. The potential for electrolyzing water is about 1.23 V (237 kJ/mol) (25 °C, 1 bar), but if there is no external heat source, 1.48 V (286 kJ/mol) is required [3]. The reaction rate using an IrO_2_ catalytic electrode increases by more than a factor of four when the temperature is raised from 80 to 120 °C, and the Nernst voltage drops by 22 mV [5]. Furthermore, if the electrolyte membrane is thermally and chemically stable, the resistance decreases with an increase in the temperature in humidified states due to the Arrhenius equation, meaning the conductivity should increase. For the entire cell, the overvoltage is reduced due to improvements in the activity of the catalyst electrode, the conductivity of the electrolyte membrane, and the reduction of the interfacial resistance between the electrolyte membrane and the catalyst electrode. Thus, efficiency improves.

At elevated temperatures (>100 °C), for PEMWE, Nafion [22,23,24,25,26], Nafion/SiO_2_ [22,27], Aquivion [28], and Nafion/PBI [23] membranes are used as electrolyte membranes. IrO_2_ is normally used as the catalyst electrode for oxygen evolution, and Pt/C is normally used as the hydrogen evolution catalyst electrode. Oxygen evolution catalyst electrodes are prepared by using a slurry of IrO_2_ and an ionomer [22,23,27], coating IrO_2_ on Ti felt [24], coating IrO_2_ on a membrane [25], or electrodepositing IrO_2_ on carbon paper [26]. In addition, Ti, which is stable even in highly oxidizing environments, is used for the carbon gas diffusion layer (GDL) and current collector on the oxygen evolution side [4,25].

In this study, as the anode electrode, we used porous IrO_2_/Ti/IrO_2_ catalyst electrodes (hereafter referred to as porous IrO_2_ catalyst electrodes) in which the IrO_2_ catalyst was coated on both sides of three types of porous Ti powder sheets with different surface treatment conditions. It was speculated that the three-dimensionally coated IrO_2_ catalyst electrode would be a useful method for improving catalytic performance and system construction for water electrolysis. As the cathode electrode, we used a Pt/C catalyst electrode coated on carbon GDL. A Nafion115 membrane was sandwiched between the porous IrO_2_ catalyst electrode and the Pt/C carbon electrode, and membrane electrode assemblies (MEAs) were prepared by using a hot press method. Using a single cell and a homemade evaluation device for elevated temperature water electrolysis, current–voltage measurements and electrochemical impedance spectroscopy (EIS) were performed at atmospheric pressure and cell temperatures in the range of 80–150 °C. In addition, the structural stabilities of the Nafion and Aquivion membranes at elevated temperatures were evaluated by monitoring the changes over time from room temperature to 150 °C and then back to room temperature using SAXS. To the best of our knowledge, we are the first to measure variable-temperature SAXS in situ up to 150 °C using Nafion and Aquivion membranes.

## 2. Experimental

### 2.1. Electrodes

Three types of porous IrO_2_/Ti/IrO_2_ catalyst electrodes prepared by coating IrO_2_ (7.5 mg/cm^2^) on both sides of Ti powder porous sheets (thickness = 0.5 mm, porosity = 57–61%) under different surface treatment conditions were obtained from Gunma, Japan Calit Co., Ltd. (IrO_2_ was considered to be three-dimensionally coated on the porous Ti powder sheet. The porous IrO_2_/Ti/IrO_2_ catalyst electrode is called a porous IrO_2_ catalyst electrode. As the electrode for hydrogen evolution, a Pt/C catalyst electrode (EIWA Co., Ltd., Tokyo, Japan) obtained by applying 0.3 mg/cm^2^ of Pt on carbon GDL (Sigracet^®^ 25BC of SGL Group Co. Ltd., Japan) was used. MEAs were obtained by hot pressing at 130 °C and 9.8 kN for 20 min.

### 2.2. Membranes

Nafion (equivalent weight, EW = 1000 g/mol) and Aquivion (equivalent weight, EW = 790 g/mol) electrolyte membranes were purchased from EIWA Co. Ltd. The electrolyte membranes were treated with boiling water (2 h), 1 M H_2_O_2_ (80 °C, 2 h), 1 M H_2_SO_4_ (80 °C, 2 h), and boiling water (2 h) before use.

### 2.3. Experimental Setup and Test Procedure

To evaluate water electrolysis, a single cell consisting of an Al end plate of 8.8 × 8.8 cm and a carbon current collector plate of 6 × 6 cm with a channel of 2.2 × 2.2 cm was used. The homemade evaluation system consisted of a part that pumped water to the anode side, an oven that controlled the temperature of the cells, and a part that controlled electrochemical equipment with a personal computer. The MEA produced by hot pressing was assembled into a single cell and then set in the evaluation system. The structure of the single cell MEA was basically the same as in previous references [3,16,26]. The water on the anode side was heated in the range of 80–100 °C using an oil bath. A water circulator was used on the anode side to supply water at a rate of 2.0 mL/min using a pump. Water was not supplied to the cathode side. The pressure on both sides of the cell was set to atmospheric pressure. The cell temperatures used in this study were 80, 100, 120, and 150 °C. Regarding the cell temperature, since the cell is placed in the oven, the set temperature of the oven was considered as the cell temperature. On the anode side, water at 80 °C was supplied when the cell was at the same temperature, and water at about 100 °C was supplied when the cell was in the range of 100–150 °C. For electrochemical measurements, the current–voltage and EIS characteristics were determined using a 1280C electrochemical test system (Solatron analytical, Japan) with a 20A booster (Tyoyo Co., Japan). The applied voltage was in the range of 1.4–1.8 V, and the current was measured while sweeping the voltage at a rate of 10 mV/s. The data were measured 2–3 times under these conditions, and values with stable current–voltage characteristics were used. EIS was measured at 1.5 V and in a frequency range of 1 Hz–20 kHz. In addition, the current characteristics over time at a cell temperature of 120 °C and 1.7 V were determined.

### 2.4. Electrical Characteristics of the IrO_2_ Electrodes

The electrical characteristics of the porous IrO_2_ catalyst electrode were measured by using a four-probe method with the PSP electrode Loresta-GX (Nittoseiko Analytech, Yamato, Japan).

### 2.5. Surface Characteristics of the IrO_2_ Electrodes

The surface characteristics of the porous IrO_2_ catalyst electrode were observed by using a field emission scanning electron microscope (FE-SEM, JSM-6700F, JEOL, Japan).

### 2.6. SAXS

The stabilities of Nafion and Aquivion membranes at elevated temperatures were measured by using SAXS (beamline BL15A2 of the Photon Factory in KEK, Tsukuba, Japan). Details of the SAXS device setup conditions can be found in a previous report [29]. The temperature was raised to 30, 80, 120, and 150 °C at a rate of 10 °C/min, and then the cell was cooled from 150 to 30 °C. The measurements were done after waiting for 10–15 min at the target temperature.

## 3. Results and Discussion

### 3.1. Characteristics of the Porous IrO_2_ Catalyst Electrodes

**Figure 1:**
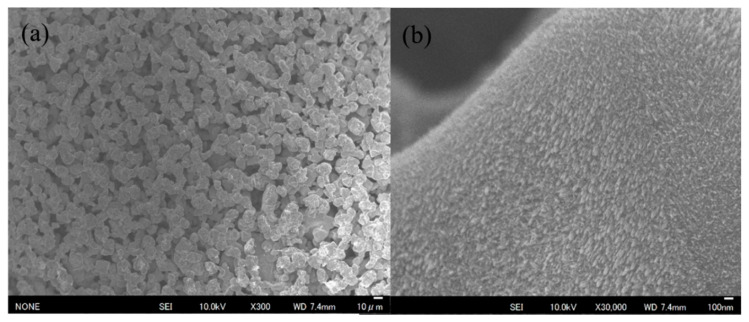

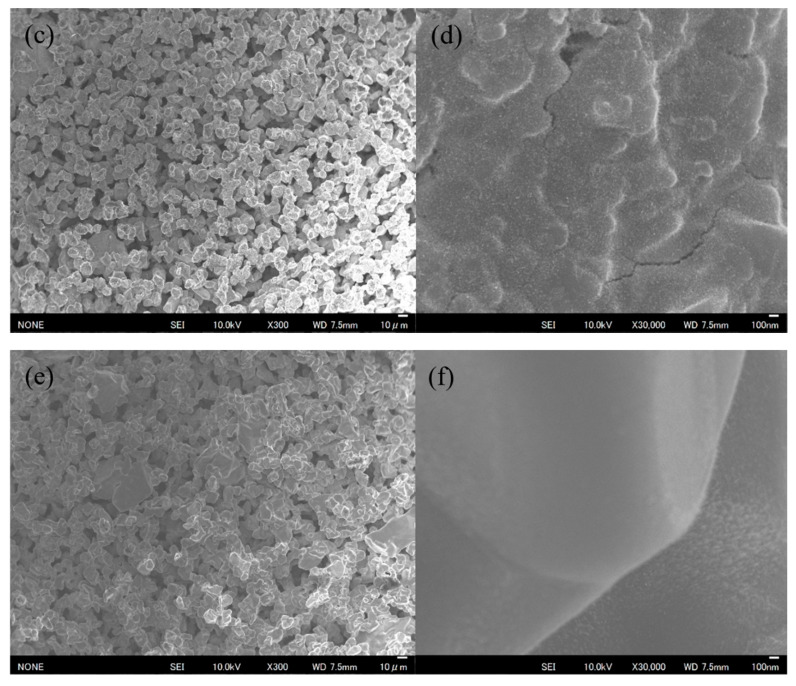
SEM images of the IrO_2_ catalyst electrodes coated on porous Ti power sheets: (**a**,**b**) sample 1, (**c**,**d**) sample 2, and (**e**,**f**) sample 3.

Various methods have been reported to improve the assembly of the oxygen evolution catalyst IrO_2_ and the membrane [22,23,24,25,26,27,28]. However, PFSA ionomers [17], which have low glass transition temperatures, and carbon [8], which is unstable at high overvoltages, are not suitable for the oxygen evolution side of elevated temperature water electrolysis cells. On the other hand, methods wherein the catalyst is directly coated on the membrane and on porous Ti GDLs are considered to be promising for improving the durability of the cell. Table 1 shows the electrical characteristics of the three types of porous IrO_2_ catalyst electrodes measured by using a four-probe method. The electrical measurements of the three samples are expressed as surface resistance (sheet resistance, ohm/sq.), volume resistivity (ohm·cm), and conductivity (S/cm). There were no significant differences in the volume resistances and conductivities of the three electrodes. The resistance of bulk IrO_2_ was 5 × 10^–5^ ohm·cm [30], and the resistances of the porous IrO_2_ catalyst electrodes (3–5 × 10^–4^ ohm·cm) were an order of magnitude higher than that of bulk IrO_2_. On the other hand, the surface resistance value of sample 1 was larger than those of samples 2 and 3. The differences in the surface resistances were thought to be related to the surface structures of the IrO_2_ coated on the porous Ti powder sheets, and therefore, SEM was performed (Figure 1). In low-magnification images (Figure 1a,c,e), IrO_2_ particles of about 10 mm were observed with large Ti powder particles below them. In addition, the surface morphologies of samples 1 and 2 appeared more uniform than that of sample 3. Furthermore, the differences in the surface structures were clear at high magnification. Sample 1 had a uniform morphology (Figure 1b) with a nanostructure, sample 2 appeared as an undefined mass (Figure 1d), and sample 3 had a morphology with severe unevenness (Figure 1f). The differences in the IrO_2_ morphologies are thought to be related to the surface treatment conditions for the Ti powder porous sheets before coating with IrO_2_.

### 3.2. Elevated Temperature Water Electrolysis

**Figure 2:**
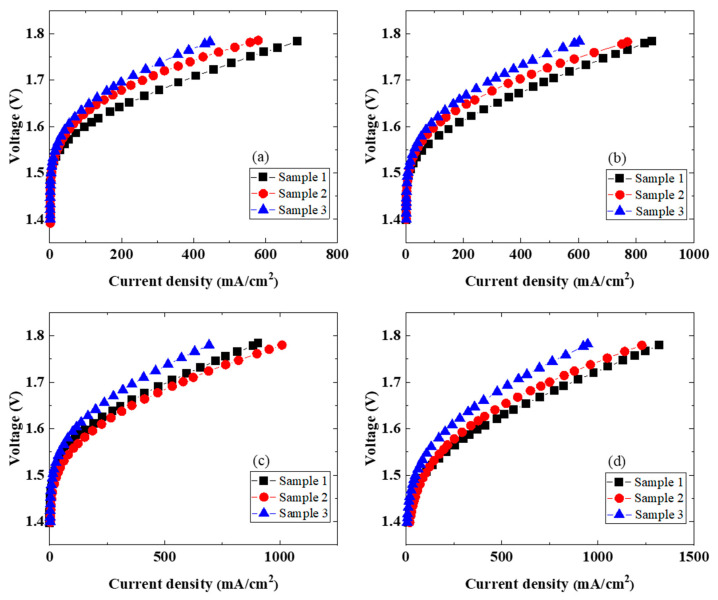
Polarization curves at different operation temperatures using the three IrO_2_ catalyst electrodes (samples 1–3): (**a**) 80, (**b**) 100, (**c**) 120, and (**d**) 150 °C.

**Figure 3:**
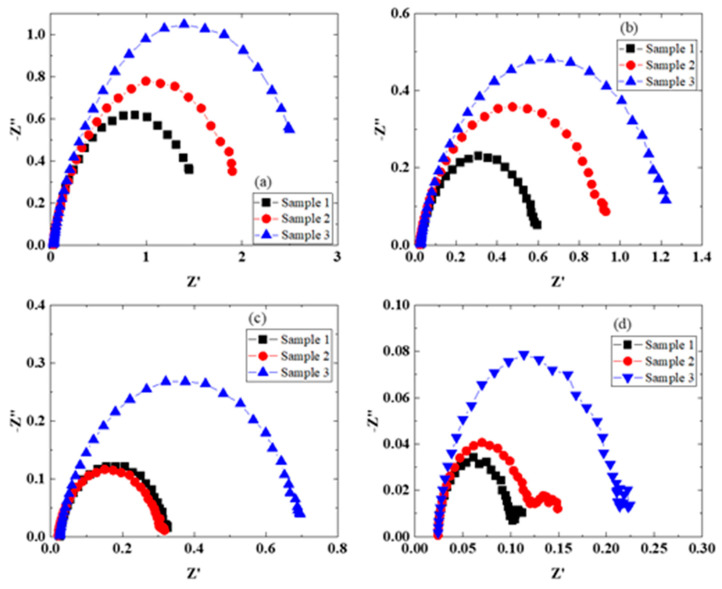
EIS properties at different operating temperatures for samples 1–3: (**a**) 80, (**b**) 100, (**c**) 120, (**d**) 150 °C.

The characteristics of elevated temperature water electrolysis were determined by using MEAs with a Nafion115 membrane, a Pt/C catalyst electrode, and the three porous IrO_2_ catalyst electrodes (Figure 2 and Figure 3). On the anode side, water at 80 °C was supplied to the cell at the same temperature, and water at about 100 °C was supplied to the cell in the range of 100–150 °C. Water liquid and vapor were both present when a cell was over 100 °C. The current–voltage characteristics depended on the porous IrO_2_ catalyst electrode (Figure 2). The current density increased with an increase in the cell temperature, and the magnitude of the increase followed the order sample 1 >sample 2 >sample 3 (Table 2). According to the values in Table 2, the voltage ranges were 10–80 mV for Sample 1 (1.57 V ➔ 1.55 V ➔ 1.54 V ➔ 1.46 V), 20–70 mV for Sample 2 (1.59 V ➔ 1.57 V ➔ 1.52 V ➔ 1.45 V), and 20–60 mV for Sample 3 (1.61 V ➔ 1.58 V ➔ 1.56 V ➔ 1.50 V). This is due to improvements in the catalyst electrode activity [21,22,28] and the conductivity of the electrolyte membrane and the decrease in the interfacial resistance (Figure 3 and Table 3) by increasing the operating temperature. In some reports, water is supplied to both sides of the cell [25,27], whereas in others, it is supplied to only the anode side [22,24,26]. At the same time, in some cases, steam, instead of liquid water, is supplied to the anode side [28]. Regarding the pressure, some cells are operated at atmospheric pressure [23,28], whereas others are operated under pressure [22,24,25,26,27]. The characteristics strongly depend on the evaluation method, how water is supplied to the cell, and the application of pressure to both sides, and these factors affect the efficiency and safety. Supplying steam has been reported by Bjerrum et al. to be good [23,28], but it is necessary to suppress the increase in the resistance because the membrane becomes dry [28]. Operating at temperatures of about 100 °C where water liquid and vapor are both present, as in this study, is also considered to be good. However, the EIS characteristics at 150 °C (Figure 3d) show that the resistance and an electric double layer capacitance are higher even on the hydrogen evolution side because the membrane becomes dry.

**Figure 4:**
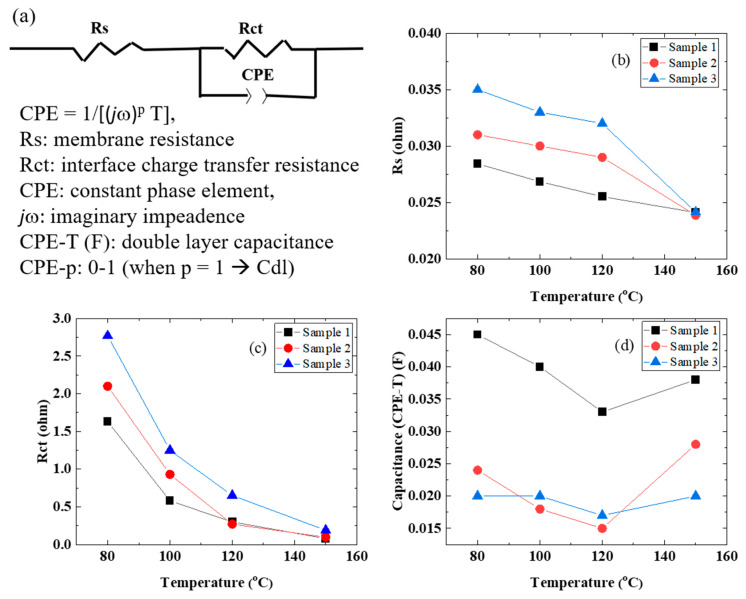
(**a**) The equivalent circuit used to fit the EIS data. (**b**) Ohmic resistance (Rs), (**c**) charge transfer resistance (Rct), (**d**) double layer capacitance (CPE-T) vs. temperature.

The EIS plot in Figure 3 was fitted using the electric circuit model in Figure 4a, affording the resistance (Rs), interfacial charge transfer resistance (Rct), and constant phase element (CPE) of the electrolyte membranes. The results are summarized in Table 3. In addition, Figure 4b–d shows the temperature dependences of Rs, Rct, and CPE, respectively. The Rs and Rct values of the electrolyte membrane decreased with an increase in the cell temperature. On the other hand, the double layer capacitance (CPE-T) clearly decreased with an increase in the cell temperature up to 120 °C and then only slightly increased up to 150 °C. Since water at about 100 °C was supplied to the anode side without pressurization, the electrolyte membrane may have dried at that cell temperature. In addition, it is thought that water liquid and vapor and oxygen bubbles were present at the interface between the membrane and the porous IrO_2_ catalyst electrode, and that the membrane dried as the temperature increased. Therefore, CPE-T tended to be higher at a cell temperature of 150 °C. The characteristics of sample 1 are the best among the electrodes used. This fact indicates that the nanostructure of the IrO_2_ catalyst (Figure 1b) is very effective [6,7,8,10,11,21].

**Figure 5:**
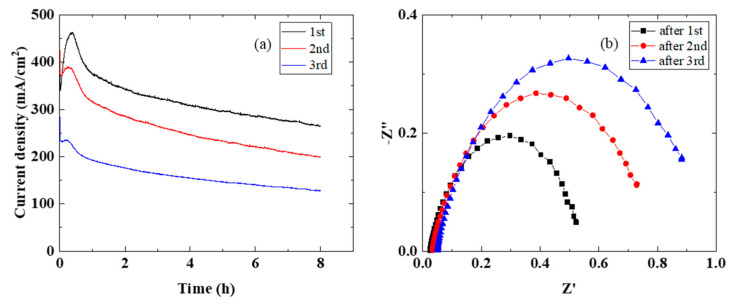
Time dependence of the single cell using sample 1 at 120 °C and 1.7 V: (**a**) current density vs. time and (**b**) EIS properties.

In order to evaluate the time dependence of elevated temperature water electrolysis, an MEA cell containing sample 1 was used. Figure 5a,b shows the characteristics of the current density over time and EIS, respectively, measured three times for 8 h at a cell temperature of 120 °C and a cell voltage of 1.7 V. The current density decreased with time over the three runs. In addition, the resistance of the membrane and Rct increased with time over the three runs. From the EIS results, the decrease in the current density over time was due to the decrease in membrane conductivity and the increase in Rct. It is thought that water liquid and vapor and oxygen were present on the anode side and that the membrane became dry and bubble layers formed in the microscopic part, resulting in a decrease in the current density and an increase in the cell resistance [16,31,32]. The performance and stability of water electrolysis largely depends on the interface between the membrane and the catalyst electrode [20,21,24,33]. In order to improve the performance and durability of water electrolysis, an ionomer can be added to the IrO_2_ catalyst electrode or coated on an electrolyte membrane [4,14,15,20,21,33]. However, it has been reported that a porous IrO_2_ catalyst electrode sheet affords similar results to ours [24]. The durability should increase if the interface between the porous IrO_2_ catalyst electrode and the membrane and the mass transfer of water liquid and vapor and oxygen are improved. On the other hand, from a study investigating the deterioration of the electrolyte membrane in fuel cells and water electrolysis [34], the deterioration rate of the membrane in water electrolysis is faster than it is in fuel cells, and at 150 °C, deterioration progresses 15 times faster than it does at 80 °C. Therefore, deterioration of the electrolyte membrane must be considered.

### 3.3. SAXS Characteristics of PFSA Membranes

**Figure 6:**
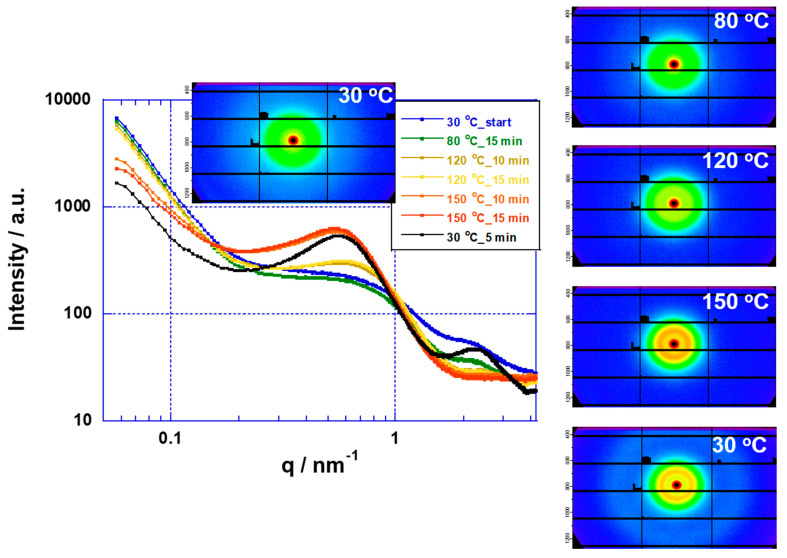
SAXS profiles for Nafion membrane at various temperatures.

**Figure 7:**
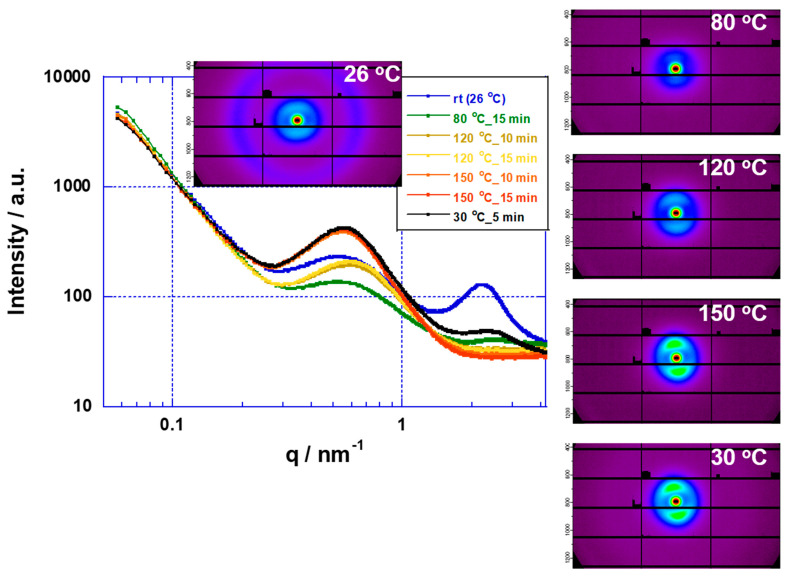
SAXS profiles of Aquivion membrane at various temperatures.

Nafion and Aquivion membranes with high chemical stabilities are used in a wide range of applications, such as fuel cells and water electrolysis. Moreover, they have been used in the study of high temperature water electrolysis above 100 °C, as in this study and previous reports. However, the glass transition temperatures of the fluorine-based electrolyte membranes (H^+^ form) are in the range of 90–120 °C [17], and their applications above 100 °C have stability problems [35,36]. Alberti, Casciola, Narducci, et al. are developing electrolyte membranes that are stable above 100 °C by using heat treatment of solutions containing Nafion and Aquivion membranes [36,37,38,39,40]. On the other hand, the conductivity measured by using atomic force microscopy (AFM) from 90 to 120 °C for up to 150 h using a Nafion membrane decreases due to changes in the conduction path as the membrane morphology changes [41]. In addition, it has been reported that the changes in the conduction properties are irreversible under high humidification conditions (RH = 100%) in the temperature range of 40–180 °C using a Nafion membrane [42]. Heat treatment above the glass transition temperatures of the membranes causes structural changes in the ionomer of the electrolyte membrane [42]. We investigated the thermal stabilities of Nafion and Aquivion membranes above their glass transition temperatures using SAXS. The SAXS analysis of Nafion membranes is described in detail in previous literatures [17,43,44]. Figure 6 and Figure 7 show scattering profiles and images using Nafion and Aquivion membranes, respectively. The polymer domain structures of Nafion and Aquivion membranes were stable from room temperature to 80 °C, except in the peak change region (q > 1), due to structural changes in the ionic clusters. However, upon raising the temperature from 80 to 150 °C, the crystal domain (q < 1) of the polymer structure grew. In the case of the Aquivion membrane, the crystal domain became anisotropic. Furthermore, when the temperature was decreased from 150 to 30 °C, the polymer did not recover its original structure. These results indicate that the polymer crystal structures of the Nafion and Aquivion membranes change above 120 °C. The SAXS method has been shown to be useful for investigating the high temperature stability of membranes. Moreover, our results are consistent with the literature in which the polymer structure of the electrolyte membranes have been reported to change on the basis of conductivity measurements above the glass transition temperature [41,42]. In addition, PFSA membranes with a low glass transition temperature (<80 °C) undergo a fatal breakdown at 120 °C due to changes in the crystal domain of the polymer [35].

### 3.4. The Relationship between the SAXS Results for Membranes and the MEA Performance Results

The SAXS results of the Nafion membrane showed a tendency for phase separation as the crystallinity increased with increasing temperature. On the other hand, in the water electrolysis characteristics of the MEA using a Nafion membrane, the conductivity of the membrane increased as the temperature increased (Figure 3). However, in the time dependence at 120 °C, the conductivity of the membrane in the MEA decreased with time (Figure 5). Although it is difficult to directly link the SAXS results with the water electrolysis properties, the decrease in the membrane conductivity over time at 120 °C may be produced by changes in the polymer structure. Of course, it is also possible that the sulfone group of the polymer is eliminated. From the above, it is possible to evaluate water electrolysis at 100 °C or higher using a Nafion membrane at the experimental level, but it is considered that there are major problems in the polymer structure and the stability of the sulfone group as an application membrane for practical use. In the future, we plan to study the stability of sulfone groups. On the other hand, the SAXS results suggested that the Aquivion membrane, which is more stable than the Nafion membrane, also has problems in its application as an elevated temperature electrolyte membrane. In other words, elevated temperature water electrolysis requires that the electrolyte membranes have high glass transition temperatures (about 200 °C) and high proton conductivities.

## 4. Conclusions

Elevated temperature water electrolysis at cell temperatures up to 150 °C was evaluated using three types of porous IrO_2_ catalyst electrodes (samples 1–3) and a Nafion115 membrane. The surface resistance of sample 1 was higher than those of samples 2 and 3, but the water electrolysis characteristics had high current densities attributed to the nanostructure. On the other hand, the electrolytic voltages and current densities differed depending on the type of porous IrO_2_ catalyst electrode. However, when the cell temperature was increased, the electrolytic voltage decreased and the current density increased. In addition, the structural stabilities of Nafion and Aquivion membranes at high temperatures were investigated by using SAXS. The polymer structures of the Nafion and Aquivion membranes were stable up to 80 °C, but above 120 °C, the crystal domain grew, and the structures did not return to the initial polymer structure after cooling. Elevated temperature water electrolysis showed high performance due to the reduced overpotential using the cell. On the other hand, electrolyte membranes with high glass transition temperatures (about 200 °C) and high proton conductivities are required.

## Figures and Tables

**Table 1:** Electrical properties of the IrO_2_ catalyst electrodes coated on porous Ti power sheets.

Sample	Sheet Resistance	Volume Resistivity	Conductivity
	(ohm/sq.)	(ohm·cm)	(S/cm)
1	1.057 × 10^–2^	5.180 × 10^–4^	1.931 × 10^–3^
2	6.295 × 10^–3^	3.116 × 10^–4^	3.209 × 10^–3^
3	7.181 × 10^–3^	3.562 × 10^–4^	2.807 × 10^–3^

**Table 2:** Current–voltage properties at different operating temperatures using different IrO_2_ catalyst electrodes.

Temp (°C)	Sample	Voltage (V) at 50 mA/cm^2^	Current Density (mA/cm^2^) at 1.8 V
80	1	1.57	689
	2	1.59	580
	3	1.61	446
100	1	1.55	856
	2	1.57	770
	3	1.58	603
120	1	1.54	906
	2	1.52	1010
	3	1.56	693
150	1	1.46	1319
	2	1.45	1229
	3	1.50	948

**Table 3:** Parameters obtained from EIS data (Figure 3) fitted to the equivalent circuit shown in Figure 4a.

Temp. (°C)	Sample	Rs (ohm)	Rct (ohm)	CPE-T (F)	CPE-p
80	1	0.028	1.64	0.045	0.80
	2	0.031	2.10	0.024	0.80
	3	0.035	2.77	0.020	0.82
100	1	0.027	0.58	0.040	0.85
	2	0.030	0.93	0.018	0.85
	3	0.033	1.25	0.020	0.83
120	1	0.026	0.30	0.033	0.87
	2	0.029	0.27	0.015	0.90
	3	0.032	0.65	0.017	0.86
150	1	0.024	0.08	0.038	0.88
	2	0.024	0.10	0.028	0.87
	3	0.024	0.19	0.020	0.87
